# The EZ-blocker for one-lung ventilation in patients undergoing thoracic surgery: clinical applications and experience in 100 cases in a routine clinical setting

**DOI:** 10.1186/s13019-018-0767-9

**Published:** 2018-06-25

**Authors:** Andreas Moritz, Andrea Irouschek, Torsten Birkholz, Johannes Prottengeier, Horia Sirbu, Joachim Schmidt

**Affiliations:** 1 Department of Anesthesiology, University Hospital of Erlangen, Krankenhausstrasse 12, 91054 Erlangen, Germany; 2 Department of Anesthesiology, University Hospital of Erlangen, Krankenhausstrasse 12, 91054 Erlangen, Germany; 3 Department of Anesthesiology, University Hospital of Erlangen, Krankenhausstrasse 12, 91054 Erlangen, Germany; 4 Department of Anesthesiology, University Hospital of Erlangen, Krankenhausstrasse 12, 91054 Erlangen, Germany; 5 Department of Thoracic Surgery, University Hospital of Erlangen, Krankenhausstrasse 12, 91054 Erlangen, Germany; 6 Department of Anesthesiology, University Hospital of Erlangen, Krankenhausstrasse 12, 91054 Erlangen, Germany

**Keywords:** One-lung ventilation, Bronchial blocker, EZ-blocker, Thoracic surgery

## Abstract

**Background:**

In certain clinical situations the insertion of a double-lumen tube (DLT) for one-lung ventilation (OLV) is not feasible or unfavorable. In these cases, the EZ-Blocker (EZB) may serve as an alternative. The aim of our analysis was to report on the clinical applications and our experience with the EZB for one-lung ventilation in 100 patients undergoing thoracic surgery.

**Methods:**

All anesthetic records from patients older than 18 years of age undergoing general anesthesia in the department of thoracic surgery with intraoperative use of an EZB for OLV at the University Hospital of Erlangen in four consecutive years were analyzed retrospectively.

**Results:**

Most frequently, EZB was used in difficult airway (27%) and for surgical procedures with high risk for left recurrent laryngeal nerve injury (21%), followed by application in intubated (12%) or tracheostomized (11%) patients. 11% of the patients had an increased risk of gastric regurgitation. Almost all EZBs were placed free of complications (99%). Clinically sufficient lung collapse was achieved in all patients. No serious airway injuries or immediate complications were documented.

**Conclusions:**

The EZB is an efficient, easy-to-use and safe airway device and enables OLV in several clinical situations, when conventional DLTs are not feasible or less favorable. Three major applications were depicted from the data: expected difficult airway, surgical procedures with necessity of intraoperative recurrent laryngeal nerve monitoring and already intubated or tracheostomized patients.

## Background

Thoracic surgery often requires lung separation and one-lung ventilation (OLV) to perform certain surgical procedures and to provide optimal site exposure. The most commonly used device is the double-lumen tube (DLT) [1]. However, the DLT is more rigid and has a larger outer diameter compared with a single-lumen tube (SLT). The placement of a DLT for one-lung ventilation may be technically difficult and has an increased risk for trauma to the trachea and the mainstem bronchi [2, 3]. Therefore the DLT should be avoided for rapid sequence induction. It is not feasible in patients with a difficult airway or tracheostomy, in patients who require unplanned OLV during an ongoing surgery or who might need prolonged mechanical ventilation after surgery, including already intubated critically ill patients [4]. To achieve lung isolation in these settings, bronchial blockers (BBs), such as Cohen Flex-tip Blocker (Cook Critical Care, Bloomington, IN) [5], the Univent Torque Control Blocker (Vitaid, Lewiston, USA) [6] and the wire-guided Arndt Endobronchial Blocker (Cook Critical Care, Bloomington, USA) [7] can be used.

The BB is a balloon-tipped semirigid catheter and can be positioned bronchoscopically into a bronchus through the inner diameter of a single-lumen tube (SLT) via a multiport adapter. It allows lung collapse distal to the occlusion. BBs cause less postoperative sore throat and hoarseness compared with DLTs [2, 8]. Campos and Kernstine demonstrated that for elective thoracic surgery the efficacy to achieve lung isolation is comparable between the DLT and the BB [9]. However, potential disadvantages include longer placement time and difficulties in device positioning, higher incidences of dislocation during surgical manipulation and very limited suctioning through the blocker [10, 11]. Another disadvantage of the BB is the difficulty of alternating OLV to either lung for bilateral procedures.

**Fig. 1:**
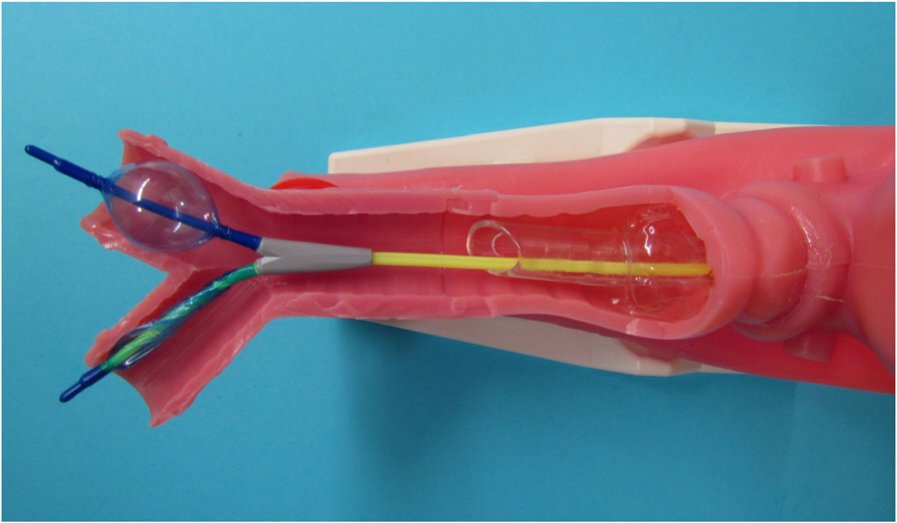
Close-up view of the EZB placed through a single-lumen tube in a manikin. The Y-shape of the distal portion facilitates the anchorage of the blocker to the carina. The two distal extensions are colored differently, both with an inflatable cuff and a central lumen. One of the polyurethane high-pressure balloons is inflated, allowing lung collapse distal to the occlusion

**Fig. 2:**
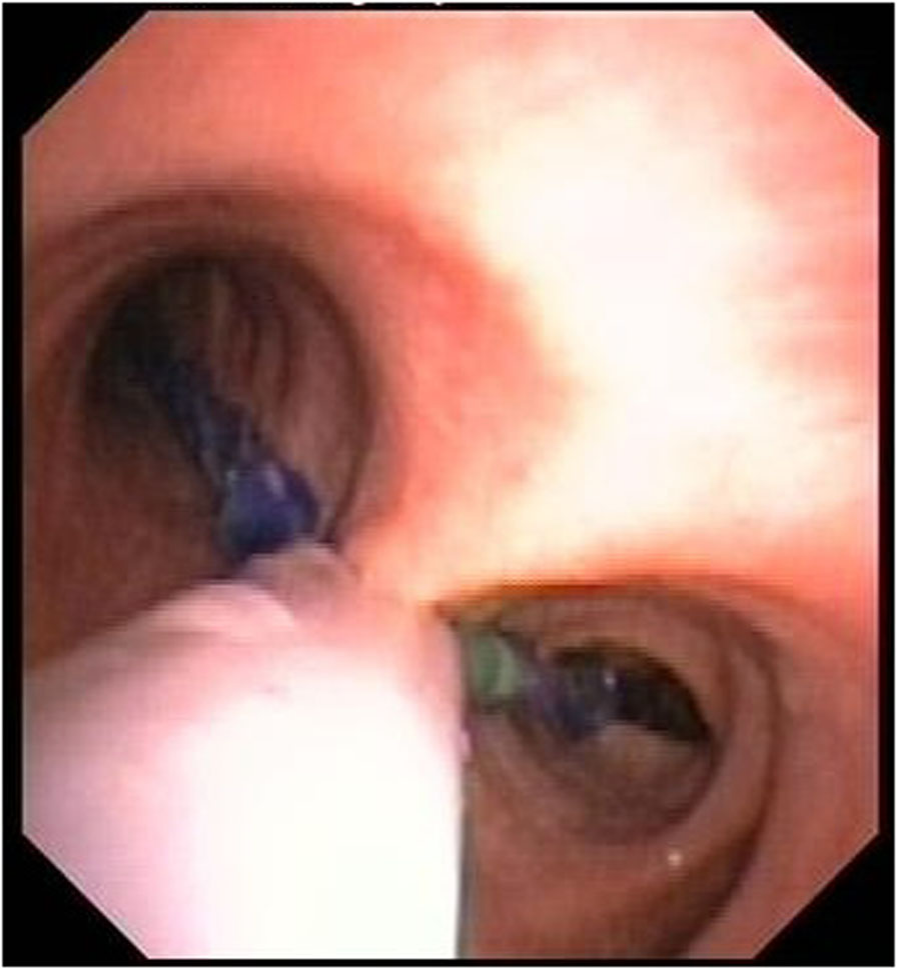
Bronchoscopic view of an EZB situated in the trachea and bronchi of a patient. The differently colored extensions are positioned in the left and the right mainstem bronchi

The EZ-Blocker (EZB; AnaesthetIQ, Rotterdam, The Netherlands) (Fig. 1), a 7-French, 75-cm, 4-lumina Y-shaped semirigid endobronchial blocker, combines some of the advantages of the DLT and the BB. This device has two different colored distal extensions, both with an inflatable cuff and a small central lumen. Two pilot balloons at the proximal part of the device serve to inflate/deflate the cuffs. Two additional lumina are available for suction or oxygen insufflation. The EZB is inserted through the designated port on the enclosed multiport adapter attached to a conventional SLT (minimum 7 mm inner diameter (I.D.)). The multiport adapter is designed to connect to a ventilation device and contains two additional upper ports, one for the blocker itself and the other for the bronchoscope. The blocker is introduced and positioned under direct bronchoscopic vision with the extensions in the left and the right mainstem bronchi (Fig. 2). However, proper deployment of the Y-shaped distal part requires a minimum of 4 cm distance between the distal end of the SLT and the carina. The Y-shape of the distal portion allows the blocker to anchor on the carina. Thus, the EZB is less prone to secondary malposition than other devices [10, 12]. The cuffs can be inflated separately, allowing OLV in either lung during the same procedure [13].

Rispoli and colleagues demonstrated that the EZB can also be used via tracheostomy [14]. Furthermore, in combination with an electromyographic endotracheal tube system (NIM EMG Endotracheal Tube, Medtronic Xomed, Jacksonville, FL) the EZB, as well as other BBs, enables recurrent laryngeal nerve monitoring during single lung ventilation [15]. This method is routinely used in our hospital in surgical procedures with necessity of OLV and high risk for left recurrent laryngeal nerve (RLN) injury.

Only a few clinical reports have assessed routine clinical performance of the EZB. In the present analysis we therefore report on the clinical applications and our experience with the intraoperative use of the EZB for OLV in 100 patients undergoing thoracic surgery in a routine clinical setting.

## Methods

All anesthetic records from patients older than 18 years of age undergoing general anesthesia in the department of thoracic surgery with intraoperative use of an EZB for OLV at the University Hospital of Erlangen in four consecutive years (January 2009 to December 2012) were analyzed retrospectively. The analysis included patient demographics, Mallampati score, Cormack and Lehane (CML) classification, surgical procedure, site of surgery, time-span of clinical experience of the responsible anesthesiologist, anesthesia drugs used for induction and maintenance, airway management, type of airway devices used, indications for the use of an EZB, difficulty with EZB placement, adequacy and duration of OLV, incidence of EZB dislocation and bronchial injury, possible decrease of oxygen saturation, ventilation parameters, need for postoperative ventilation and finally adverse events during anesthesia. The data were retrieved from the electronic patient data management system (NarkoData; IMESO, Hüttenberg, Germany). The indications for the use of an EZB were recorded at the time of insertion. The adequacy of lung collapse was clinically assessed by the thoracic surgeon. All available data were anonymized and transferred to an Excel datasheet (Microsoft, Redmond, USA).

The University Hospital of Erlangen provides the full spectrum of thoracic surgery. For thoracic anesthesia with OLV standard operation procedures (SOPs) were well established and remained unchanged during the study period. The SOPs included induction and maintenance of anesthesia, as well as the clinical management of OLV and the use of different devices for OLV (DLT, EZB, BB). Standard monitoring for thoracic surgery with OLV included electrocardiogram, pulse oximetry, capnography and invasive blood pressure (SC9000XL System; Siemens, Erlangen, Germany). The DLT represents the standard device for OLV. During the reported time period, the Arndt Endobronchial Blocker and the EZB served as an alternative. EZB use in routine clinical practice was provided as follows: After induction of anesthesia and insertion of a SLT (Rüschelit Super Safety Clear, Rüsch GmbH, Kernen, Germany; Mallinckrodt Lo-Contour Oral/Nasal Tracheal Tube Cuffed Reinforced, Covidien, Tullamore, Ireland; in case of RLN monitoring: NIM EMG Endotracheal Tube, Medtronic Xomed, Jacksonville, FL) the patients were placed in a right or left decubitus position for the surgical procedure. After verifying under direct bronchoscopic vision that the distal end of the SLT is at least 4 cm above the carina, the EZB was lubricated with silicone spray and introduced through one of the two proximal ports of the multiport adapter with its cuffs completely deflated. Further advance was guided with a fiberoptic bronchoscope (FOB), placing the distal EZB ends into the right and left mainstem bronchi under direct bronchoscopic vision. If there was less than 4 cm distance between the distal end of the SLT and the carina, the SLT was retracted more proximal. With the EZB finally properly placed, the SLT was readvanced into the trachea as necessary. Both movements required a deflated SLT cuff. To test the bronchial sealing, the cuff of the EZB was inflated under FOB guidance with an appropriate volume and deflated again. The insertion technique described was used for all patients from the commencement of the study period and all EZBs were placed under supervision of an attending physician in accordance with the standard operation procedure (SOP). To facilitate unilateral lung collapse, a specific sequence of action was used after the surgeon breaks the pleural vacuum: First, disconnection of the tube from the ventilator allows the operated lung to collapse. After 20 s, reinflation of the blocker cuff with the same volume of air as used before and reconnection of the tube to the ventilator establishes ventilation of the dependent lung. In case of ventilation problems as an increased peak pressure or total/subtotal ventilation of the non-dependent lung, the SOP ordered immediate FOB examination to check and eventually reposition the EZB. According to the SOP, after removing the EZB at the end of surgery, the mucosa of the tracheobronchial system was observed with the FOB for possible damage due to the EZB.

For surgical procedures with high risk for left RLN injury, e. g. resection of the left upper lobe and lymph node dissection in the aortopulmonal window, a ready-made EMG endotracheal tube system (NIM EMG Endotracheal Tube, Medtronic Xomed, Jacksonville, USA) was used for RLN monitoring during single lung ventilation [15].

In case of an expected difficult airway, awake fiberoptic intubation was performed initially via the nasotracheal route using a flexible fiberoptic bronchoscope. To prevent potential trauma to the nasal turbinates, the septum or the posterior nares, a smaller SLT (size 6.5 to 7.0 mm I.D.) was primarily inserted. However, for the EZB a SLT with a minimum I.D. of 7 mm is necessary. Moreover, sinusitis and local abscesses as a complication of nasotracheal intubation have been reported in critically ill patients who needed prolonged mechanical ventilation after surgery [16]. Thus, after induction of general anesthesia, the patients received video laryngoscopy (Glidescope, Verathon Medical, Rennerod, Germany or C-MAC Karl Storz, Tuttlingen, Germany) or flexible orotracheal fiberoptic bronchoscopy to change the airway to an orotracheal SLT of 7 mm I.D. (female patients) to 8 mm I.D. (male patients). To maintain airway access at any time, a Cook Airway Exchange Catheter (CAEC, Cook Medical Inc., Bloomington, IN) was left endotracheally in place after the nasotracheal tube has been removed. Before placement of the orotracheal tube, the CAEC was pulled out of the trachea.

In case of a difficult airway and the necessity of RLN monitoring awake fiberoptic nasotracheal intubation was primarily performed. The patients were then reintubated orotracheally with the Xomed EMG endotracheal tube size 7.0 mm I.D. (female patients) to 8.0 mm I.D. (male patients), using video laryngoscopy (Glidescope, Verathon Medical, Rennerod, Germany or C-MAC Karl Storz, Tuttlingen, Germany) or flexible orotracheal fiberoptic bronchoscopy and a CAEC.

Data were anonymized for statistical analysis. Descriptive statistical analysis was done using Statistica version 6 (StatSoft (Europe) GmbH, Hamburg, Germany). Categorical variables were given as absolute numbers and percentages of their occurrence. Continuous variables were presented as medians (interquartile range, IQR).

## Results

Over the study period the electronic data records from 100 patients undergoing general anesthesia in the department of thoracic surgery with intraoperative use of an EZB for OLV were analyzed. In the same period 1208 DLTs were used for lung isolation. Characteristic data of the patients included in the analysis and surgical procedures are summarized in Table 1.

**Table 1:** Characteristics of patients, distribution of surgical procedures and site of surgery

Patient characteristics	n (%) or median (IQR)
Gender, n (%)	
Female	32 (32)
Male	68 (68)
Age (y), median (IQR)	65.5 (53–70.5)
BMI (kg/m^2^), median (IQR)	25.3 (21.8–29)
ASA physical status, n (%)	
I	3 (3)
II	33 (33)
III	52 (52)
IV	12 (12)
Mallampati score, n (%)	
I	18 (18)
II	38 (38)
III	14 (14)
IV	6 (6)
not specified	24 (24)
CML classification, n (%)	
I	41 (41)
II	8 (8)
III	7 (7)
IV	0 (0)
not specified	44 (44)
Surgical procedures, n (%)	
VATS procedure	36 (36)
Wedge resection	12 (12)
Segment resection	3 (3)
Ligation of thoracic duct	3 (3)
Pleural decortication	3 (3)
Lobectomy	1 (1)
Other surgical procedures	14 (14)
Thoracotomy	64 (64)
Lobectomy	17 (17)
Wedge resection	12 (12)
Pleural decortication	7 (7)
Segment resection	6 (6)
Pneumonectomy	2 (2)
Bilobectomy	1 (1)
Ligation of thoracic duct	1 (1)
Other surgical procedures	18 (18)
Site of surgery, n (%)	
Right	51 (51)
Left	46 (46)
Bilateral	2 (2)
Median sternotomy	1 (1)

Data are presented as absolute number of patients (%) or as median (IQR)

The involved anesthesiologists had a median clinical experience of 5 years (IQR: 3.5–7.8) and worked under close supervision of attending physicians.

Anesthesia induction was performed with etomidate (46%), propofol (44%), thiopentone (9%) or ketamine and midazolam (1%). Fentanyl (88%), sufentanil (8%) or remifentanil (4%) were used as intravenous anesthetic agents for induction. Rocuronium (83%), cis-atracurium (10%), succinylcholine (5%) or mivacurium (1%) were used as neuromuscular blocking agents. Anesthesia was maintained by total intravenous anesthesia (TIVA) using propofol in combination with fentanyl, remifentanil or sufentanil.

After induction of general anesthesia, most of the patients with EZB for OLV were intubated orotracheally (64%). In case of difficult airway, fiberoptic bronchoscope-guided awake nasotracheal intubation was performed (13%). Tracheostomized (11%) or already intubated patients from the intensive care unit (12%) were also enrolled in this retrospective analysis. The size of the SLTs used in this study varied from 6.5 to 7.0 mm I.D. for nasotracheal and from 7.0 to 8.0 mm I.D. for orotracheal intubation. The tracheostomy tubes had an internal diameter from 8.0 mm to 10.0 mm. The mean cumulative time of OLV was 67 min (IQR: 43–85 min). During OLV, pressure-controlled ventilation (91%) or volume-controlled ventilation (9%) was used on the dependent lung.

Difficult airway (27%) or surgical procedures with necessity of OLV and high risk for left RLN injury (21%) were the most frequent preconditions. The EZB was also used in already intubated (12%) or tracheostomized (11%) patients. 11% of our patients had an increased risk of gastric regurgitation. The clinical applications of the EZB are summarized in Table 2. In one case, a DLT was not feasible because the endobronchial portion of the DLT, placed in the left mainstem bronchus, applied too much pressure to the carina. Thus, the patient had to be reintubated with a SLT and an EZB was placed for OLV.

**Table 2:** Clinical applications of the EZB

Documented indications	n (%)
Difficult airway	27 (27)
Oral cancer	12 (12)
Vocal cord dysfunction	4 (4)
Mediastinal mass syndrome	1 (1)
Subcutaneous emphysema	1 (1)
Limited mouth opening	1 (1)
Tracheal dislocation	1 (1)
Other reasons	7 (7)
RLN monitoring	21 (21)
Intubated patients	12 (12)
Tracheostomized patients	11 (11)
Rapid sequence induction	11 (11)
Not fasting	3 (3)
Obesity	2 (2)
Gastroesophageal reflux disease	1 (1)
Other reasons for increased risk of gastric regurgitation	5 (5)
Difficult airway and RLN monitoring	5 (5)
Rapid sequence induction and RLN monitoring	1 (1)
Other reasons	4 (4)
DLT cuff leak	1 (1)
DLT not placeable	1 (1)
DLT applied too much pressure to the carina	1 (1)
Ailing teeth	1 (1)
Medical education	8 (8)

Data are presented as absolute number (%)

No complications were reported in 99% of patients. In a single case, the EZB got stuck with the FOB inside the SLT. However, the patient was reintubated and the EZB was introduced and positioned without any problems. Clinically sufficient lung collapse was achieved in all patients. In one patient OLV had to be abandoned due to severe desaturation (oxygen saturation < 90%) on initiation of lung collapse. In two cases, the EZB had to be repositioned under FOB guidance due to lung isolation failure during surgical manipulations.

No serious airway injuries or immediate complications from EZB placement or FOB were documented. In a single patient with pleural empyema, the EZB had to be removed intraoperatively due to an unplanned bilobectomy of the right lung. The distal extension of the EZB in the right mainstem bronchus interfered with the surgical procedure and was at risk of being caught in the sutures.

92% of the patients received postoperative intensive care, whereof 29% required ventilatory support. 8% were transferred to the intermediate care unit or the thoracic surgery ward after a minimum of one hour in the anesthesia recovery room.

## Discussion

In several clinical situations, lung separation and OLV are essential. The choice of airway device for OLV depends on the experience of the anesthesiologist and the requirements of the surgical procedure. The DLT is still the most commonly used device to enable single lung ventilation [1]. However, in some situations conventional DLTs for one-lung ventilation are not feasible [4]. In these cases, a BB or an EZB serve as valuable alternatives. There have been relatively few reports that have assessed the effectiveness of the EZB in a routine clinical setting. It has been shown that the EZB can be positioned quickly and easily during OLV [17, 18]. As previous reports mainly focused on the performance of the device, the aim of our analysis was to report on the clinical applications and our experience with the EZB in a routine clinical setting.

The successful use of the EZB has been reported in patients with a difficult airway or tracheostomy, in those with increased risk of gastric regurgitation or who have unplanned OLV requirements during an ongoing surgery and who might need prolonged mechanical ventilation after surgery including critically ill intubated patients [4]. We could confirm these findings. In our analysis the most common indication for the use of the EZB was an expected difficult airway. The EZB enables OLV after awake fiberoptic intubation and thus improves the safety of these patients. Tracheostomized or already intubated patients were represented in almost equal numbers. As airway exchange in these critically ill patients is associated with an increased risk of clinically important procedural complications [19], BBs and especially the EZB might pose significant advantages in the management of these patients.

About one-tenth of our patients had an increased risk of gastric regurgitation. As the placement of a DLT for OLV may be technically difficult and may require too much time the EZB serves as a safe and easy alternative in patients with the necessity of a rapid sequence induction. Moreover, we could demonstrate another indication for the use of an EZB. In combination with a Xomed EMG endotracheal tube the EZB enabled recurrent laryngeal nerve monitoring during left upper lobe surgery or aortopulmonary window lymph node dissection, with high risk for left RLN injury and necessity of OLV, in twenty-one patients. The advantages of this method are as follows: RLN monitoring is possible even in cases of difficult airway or rapid sequence induction, and it allows repositioning of the SLT at any time in case of lacking EMG signal maintaining OLV [15]. RLN monitoring in patients requiring OLV could also be performed with any other BB. However, the main advantage of an EZB compared to other BBs is its Y-design, which shows similarities with the anatomic structure of the tracheobronchial tree. Based on our clinical experience the Y-shaped distal part allows the blocker to anchor on the carina and leads to positional stability. The EZB is secured between the carina and the seal at the proximal end of the tracheal tube. Thus, the two distal extensions, which are positioned in the left and the right mainstem bronchi, mutually stabilize each other by applying counter pressure on the bronchial mucosa in case of surgical manipulation. This is according to the findings of Kus and colleagues, who described that the EZB had a lower incidence of malpositioning than the Cohen Flex-Tip Blocker [12]. In addition, the Arndt Endobronchial Blocker is even less stable than the Cohen Flex-Tip and other BBs [10]. Mourisse and colleagues observed, that the EZB causes less injury to the tracheal and bronchial mucosa, when compared with the DLT. Furthermore, the quality of lung deflation is equally good and the EZB stays equally well in place during OLV [18]. Our findings confirm the positional stability. During OLV, the EZB had to be repositioned in two cases only. Kus and colleagues also demonstrated that the EZB had a shorter time to correct positioning compared with the Cohen Flex-tip Blocker [12]. Another advantage of the EZB is the possibility to alternate OLV to either lung during bilateral procedures in already intubated or tracheostomized patients. During the study period the EZB enabled OLV during bilateral VATS (Video Assisted Thoracoscopic Surgery) procedure and bilateral thoracotomy in two critically ill intubated patients. In contrast to the DLT, there is also no need to change the SLT in cases where postoperative ventilation is mandatory.

Regarding the cost-effectiveness, the EZB is almost four times more expensive than a DLT. Therefore the EZB should be used efficiently in the depicted applications. However, compared to other BBs, based on our purchase price, the EZB is marginally cheaper than the Arndt Endobronchial Blocker.

Despite of the described advantages and the almost equal cost when compared with other BBs, the EZB has some limitations which have to be mentioned. First, the EZB is initially designed for adult patients and thus only available in one size. Although extraluminal placement of an EZB can be successfully used to provide lung isolation in children down to 6 years of age [20], we use an Arndt Endobronchial Blocker for pediatric patients. The 5-French Arndt Endobronchial Blocker can be used as a consistent, safe method of single lung ventilation in most young children. The smallest tracheal tube recommended for use with this BB is 4.5 mm I.D. [21]. Second, in one case the EZB had to be removed due to an unplanned resection of the right mainstem bronchus. Thus, in some clinical situations, such as pneumonectomy or bronchial sleeve resection, where the presence of the distal tip of an EZB or any other BB would interfere with the surgical procedure or would be at risk of being stuck in the sutures, DLTs may be more suitable. Third, an EZB cannot be used for selective lobar blockade, which can be performed with other BBs. We therefor also use the Arndt Endobronchial Blocker. Fourth, a minimum of 4 cm distance between the distal end of the SLT and the carina is mandatory to permit the Y-shaped distal part to be deployed properly. Finally, another important limitation of the EZB is the smaller suction channel, when compared with a DLT. The EZB has a 7-French outer diameter, which is split into two lumens leaving a minimal diameter for each lumen. Thus, it is nearly impossible to apply any effective suction or oxygen insufflation to the nondependent lung in case of hypoxemia [22].

Vegh and colleagues found that the use of the EZB was safe and easy [23]. We can confirm these findings. No complications were reported in 99% of patients. Clinically sufficient lung collapse was achieved in all patients. However, the use of FOB guidance for initial placement and for repositioning must be considered as mandatory, but this also applies for the DLT and other BBs. No serious tracheobronchial injuries or immediate complications from EZB placement or FOB were documented. Only in one case the EZB got stuck with the FOB inside the SLT due to an overly fast FOB advancement. This could be avoided by carefully advancing the FOB in a safe distance to the Y-shaped distal part of the EZB.

The present analysis has certain limitations, mainly related to its retrospective character. First, as in every retrospective analysis, the clinical circumstances were not uniform for every case. Second, the retrospective nature of the study implies a high dependency on the quality and completeness of documentation. Finally, in this study OLV was performed by anesthesiologists with a median clinical experience of 5 years and under supervision of an attending physician. Thus, results may differ in the hands of less experienced anesthesiologists.

## Conclusions

The EZB is an efficient, easy-to-use and safe airway device to allow single lung ventilation and to provide optimal surgical exposure. The EZB combines the advantages of the DLT and the BB and enables OLV in several clinical situations, when conventional DLTs are not feasible or less favorable. Three major applications were depicted from the data: expected difficult airway, surgical procedures with necessity of RLN monitoring and already intubated or tracheostomized patients.

## Abbreviations

BB: Bronchial blocker
CAEC: Cook Airway Exchange Catheter
CML: Cormack and Lehane
DLT: Double-lumen tube
EZB: EZ-Blocker
FOB: Fiberoptic bronchoscope
I.D.: Inner diameter
IQR: Interquartile range
OLV: One-lung ventilation
RLN: Recurrent laryngeal nerve
SLT: Single-lumen tube
SOP: Standard operation procedures
TIVA: Total intravenous anesthesia
VATS: Video assisted thoracoscopic surgery
